# Implementation of a Robotic Liver Surgery Program in a Large Municipal Hospital: A Propensity Score-Matched Analysis of Clinical, Economic, and Learning Curve Outcomes

**DOI:** 10.3390/jcm15166413

**Published:** 2026-08-19

**Authors:** Daniel Eckhardt, Victoria Vaas, Teresa Colbatzky, Cornelia Porzner, Luca Giulini, Martin C. Wittmann, Markus K. Diener, Felix J. Hüttner, Patrick Heger

**Affiliations:** Department of General, Visceral and Thoracic Surgery, Klinikum Nürnberg, Paracelsus Medical University, 90419 Nuremberg, Germany; daniel.eckhardt@klinikum-nuernberg.de (D.E.); teresa.colbatzky@klinikum-nuernberg.de (T.C.);

**Keywords:** robotic liver surgery, learning curve, IWATE score, cost analysis, propensity score matching

## Abstract

**Background**: Evidence regarding implementation of robotic liver surgery outside academic centers remains limited. This study evaluated perioperative, economic, and learning-curve outcomes during implementation of robotic liver surgery in a municipal hospital. **Methods**: Consecutive liver resections performed between November 2023 and June 2025 were retrospectively analyzed from an institutional database using propensity score matching. Primary outcomes included Textbook Outcome (TO), major complications (Clavien–Dindo ≥ IIIa), operative duration, blood loss, and postoperative quality of life (QoL). Learning curves were analyzed using cumulative sum analysis adjusted for IWATE difficulty levels. An economic subgroup analysis evaluated cost components. **Results**: After matching, operative duration, TO, and major complications were similar between groups, whereas blood loss was significantly lower in the robotic group. Risk-adjusted learning curves demonstrated outcome-specific change-points, with the lowest TO rates observed at 8 cases and peak major complication rates at 29 cases. QoL did not differ between groups. Total costs were comparable. **Conclusions**: Implementation of robotic liver surgery was associated with lower blood loss, but no statistically significant differences in the other principal clinical outcomes, total costs, or QoL. Complexity-adjusted learning curves demonstrated multidimensional learning patterns with different breakpoints across evaluated outcomes.

## 1. Introduction

Centralization of hepatectomy in specialized units has improved outcomes in many countries [1,2]. However, the structured implementation of robotic liver surgery outside university centers remains challenging. Several high-volume centers have reported outcomes of robotic liver surgery, including extended learning curves [3,4,5,6,7,8,9,10,11,12,13], and current evidence suggests that both minor and major robotic hepatectomies are feasible and safe [14]. Nevertheless, there is still limited guideline-level evidence [14], and current recommendations emphasize staged implementation and the complementary use of open and laparoscopic techniques [14,15,16].

Meta-analyses indicate that robotic liver resection may reduce blood loss, morbidity, and length of hospital stay (LOHS) [17], while maintaining comparable oncological outcomes and mortality rates [14,17]. To support case selection and standardize the reporting of technical difficulty, current guidelines recommend using complexity grading systems [14,15], such as the IWATE score [18].

Despite the increasing adoption, several aspects of implementation remain insufficiently characterized. In particular, the case volume required to achieve procedural stability, the interaction between surgical complexity and learning process, and the relationship between quality outcomes and costs are not well understood [14,19]. Most studies have focused on isolated technical or clinical outcomes such as operative time and blood loss, or major complications [20,21], while composite indicators such as Textbook Outcome (TO) [22] have rarely been applied to assess learning processes [23]. In addition, the economic implications of learning and the coupling of costs and quality during implementation remain largely unexplored.

The aim of this study was to evaluate the implementation process of a robotic liver surgery program in a non-university setting by comparing perioperative, clinical, oncological, economic and quality-of-life outcomes with open liver surgery. A secondary objective was to characterize complexity-adjusted learning curves and to compare these trajectories with outcomes from corresponding open procedures.

## 2. Materials and Methods

### 2.1. Study Design and Data Collection

All consecutive liver resections performed between 1 November 2023 and 30 June 2025 were identified from our prospectively maintained institutional database. Robotic procedures were performed using the Da Vinci Xi platform (Intuitive Surgical, Sunnyvale, CA, USA). Laparoscopic approach was excluded as it was neither fully established nor part of the implementation process.

Baseline characteristics included age, sex, Body Mass Index (BMI), Charlson Comorbidity Index [24], preoperative diagnosis, and previous major abdominal surgery. Operative variables comprised surgical approach, type of resection, IWATE difficulty score/level [18], duration of surgery, and estimated blood loss. Postoperative parameters included LOHS, pathologic findings, TO [22], and complications graded according to Clavien–Dindo classification (CDC) [25]. Hepatectomy-specific complications were defined and classified according to the International Study Group of the Liver (ISGLS) criteria: posthepatectomy liver failure (PHLF) [26], bile leakage (BL) [27], and hemorrhage (PHH) [28]. Overall morbidity was quantified using the Comprehensive Complication Index (CCI) [22]. Quality of life at postoperative day 90 was assessed using the 5-level EQ 5 dimensions (EQ-5D-5L) instrument (https://euroqol.org/information-and-support/euroqol-instruments/eq-5d-5l/, accessed on 23 June 2025).

Patients undergoing surgery for perihilar cholangiocarcinoma, simple liver cysts, or cystic echinococcosis were excluded, because these patients routinely underwent open or laparoscopic surgery. The choice of surgical approach was not protocolized.

### 2.2. Statistical Analysis

Continuous variables were reported as median with interquartile range (IQR) and compared using Wilcoxon rank-sum tests. Mean differences with 95% confidence intervals (95%-CI) were additionally provided. Categorical variables were presented as counts with percentages and compared using chi-squared tests with Yates’ correction, with results expressed as odds ratios (OR) with 95%-CI. *p*-values < 0.05 were considered statistically significant.

Primary outcomes were TO, major complications (CDC ≥ IIIa), estimated blood loss, duration of surgery, and LOHS. Multivariable linear and logistic regression models were used to assess association between predictors and outcomes.

### 2.3. Propensity Score Matching

To improve comparability between treatment groups, propensity scores were constructed using age, BMI, American Society of Anesthesiologists (ASA), Charlson Comorbidity Index, IWATE Level, multivisceral resection, history of liver re-resection, cirrhosis, and major liver resection. One-to-one nearest-neighbor matching without replacement was performed using a caliper of 0.25 of the logit of the propensity score. Performance of bile duct reconstruction was used for sensitivity analysis. Covariate balance was assessed using standardized mean differences < 0.1. As residual imbalance remained for age and the Charlson Comorbidity Index, outcome analyses in the matched cohort were additionally adjusted for these covariates. Continuous outcomes were analyzed using linear regression and binary outcomes using logistic regression, with cluster-robust standard errors accounting for matched pairs.

### 2.4. Learning Curve Model

Learning curve effects were evaluated for robotic surgery. Cases were ordered chronologically by date. Learning curves were analyzed for the surgical team as a whole instead of individual surgeons’ learning curves. Four outcomes were analyzed: estimated blood loss and duration of surgery, TO, and major complications.

Risk adjustment was performed using the IWATE difficulty level as a categorical covariate. For continuous outcomes, expected values were modelled by linear regression, and learning curves were constructed as a cumulative sum (CUSUM) curves from model residuals (Σresidual). For binary outcomes, expected probabilities were estimated using logistic regression, and risk-adjusted CUSUM was computed as Σ(y − $\hat{p}$).

Segmented regression was fitted to each risk-adjusted CUSUM curve as a function of case sequence to estimate change-points (breakpoints). Breakpoint estimates with 95%-CI were obtained; where analytical confidence intervals were unstable, percentile bootstrap confidence intervals were derived. The presence of a significant change in slope was assessed using the Davies test.

### 2.5. Economic Analysis

A predefined economic subgroup including all cases with complete perioperative cost data was analyzed, because of a change in the accounting/cost data system. Data were only available until 31 December 2024 without any other selection criterion than date of surgery. Costs were categorized into intraoperative, inpatient, and treatment-related costs. Total hospital costs, reimbursement and net value were assessed. Comparisons between surgical approaches were performed using non-parametric tests.

To identify economic drivers, multivariable linear regression models were fitted using total hospital costs as dependent variable. Candidate predictors included blood loss, operative duration, IWATE score, and major complications. Stratified analyses were additionally performed according to surgical approach.

A multivariable linear regression model was constructed to identify independent determinants of net value, defined as difference between total reimbursement and total hospital costs per case. Candidate predictors were age, CCI, TO, LOHS, operative duration, intraoperative blood loss, IWATE score, surgical approach, and extent of resection. Continuous variables were retained in their original scale. Regression coefficients (β) with corresponding 95%-CI and *p*-values were reported. Coefficient estimates are reported in Euros (EUR) per unit, along with 95%-CI and two-sided *p*-values.

### 2.6. Software

All analyses were performed in R version 4.5.0 (R Foundation for Statistical Computing, Vienna, Austria) using the following packages: readxl, tidyverse, janitor, rstatix, effectsize, broom, MatchIt, WeightIt, cobalt, brms (Stan backend), bayestestR, ggplot2, openxlsx, confint, segmented, patchwork, epitools, and nls2.

## 3. Results

### 3.1. Patient Characteristics

Between 1 November 2023 and 30 June 2025, 142 liver resections were performed; 34 were excluded according to predefined criteria, leaving 108 (54 open, 54 robotic). Two robotic cases (3.7%) were converted to open surgery and analyzed according to intention-to-treat. The 90-day follow-up was complete for all included patients. All operations were performed by a team of four surgeons, only one had-prior experiences in robotic liver surgery and being present at all operations.

Baseline characteristics were largely comparable between both groups (Table 1). The Charlson Comorbidity Index was slightly lower in the robotic group (5.5 (2.00–6.00) vs. 6.00 (4.00–6.00); *p* = 0.049), while colorectal liver metastases were significantly more frequent in open resections (46.3% vs. 24.1%; *p* = 0.027).

### 3.2. Intraoperative Parameters

Types of resection and major hepatectomy were comparable (Table 2). Instead, liver re-resections were significantly more often performed via open approach (15 vs. 4 cases; *p* = 0.011), and we observed a tendency, yet not significant, towards open approach in multivisceral resections (11 vs. 5 cases; *p* = 0.176) and after major previous abdominal surgery (25 vs. 15 cases; *p* = 0.085). Bile duct reconstruction including biliodigestive anastomosis occurred in three open cases and in none of the robotic cases (*p* = 0.242). IWATE difficulty scores did not differ significantly (7 (6.00–10.00) vs. 6.50 (5.00–10.00); *p* = 0.154).

Consistent with the lower blood loss observed during robotic surgery (400 mL (150–850) vs. 100 mL (50–300); *p* <0.001), the proportion of patients requiring intraoperative transfusion were significantly lower in the robotic groups for red blood cell concentrates (24.1% vs. 3.7%; *p* = 0.0054) and fresh frozen plasma (27.8% vs. 9.3%; *p* = 0.0258). Operative duration was also significantly shorter: 209 min (160–264) vs. 166.5 min (130–216); *p* = 0.014. These findings may partly reflect differences in case selection between approaches.

### 3.3. Postoperative Outcomes

TO occurred in 37 (68.5%) open cases and 40 robotic cases (74.1%), respectively (*p* = 0.671). As shown in Table 3, overall (*p* = 0.349) and major complication rates (9 (16.7%) of open cases and 10 (18.5%) of robotic cases (*p* > 0.99)) were comparable between both approaches. There were no significant differences in liver-specific complications (PHLF, PHH or BL), although PHH and BL were markedly lower in the robotic group. R0-resection rates were also comparable: 84.6% after open liver surgery and 86.8% after robotic resection.

Three patients (5.6%) died after open and two (3.7%) after robotic resection during the 90-day follow-up: one died of septic multiorgan failure due to pneumonia, another one of grade C PHLF and kidney failure, two of septic multiorgan failure after mesenteric ischemia, and one after grade C PHH.

Postoperative LOHS was significantly shorter after robotic resection: 7 days (6–12) vs. 5 days (4–9); *p* < 0.001.

### 3.4. Predictors of Operation Duration, Estimated Blood Loss, LOHS, TO, and Major Complications

Multivariate analysis identified male sex (β = +27.5 min (2.8–52.1), *p* = 0.03), pringle maneuver (β = +33.6 min (6.4–60.8), *p* = 0.016), and multivisceral resection (β = +136.6 min (98.3–174.9), *p* <0.001) as the strongest drivers of duration of surgery.

Younger patients (β = −7.0 mL/year (−13.6–−0.4), *p* = 0.038) and those with higher BMI (β = +15.7 mL/unit (0.5–30.9), *p* = 0.043) were independent predictors of increased blood loss, while robotic resection significantly reduced blood loss (β = −321 mL (−497–−145), *p* <0.001). While male sex was associated with significantly shorter LOHS (β = −5.0 days (−9.8–−0.2), *p* = 0.040), multivisceral resection is a predictor of prolonged recovery (β = +13.5 days (6.1–21.0), *p* < 0.001). ASA class III (OR = 0.16 (0.03–0.91), *p* = 0.039) and higher Charlson Comorbidity Index (OR = 0.64 (0.45–0.91), *p* = 0.012) reduced likelihood of achieving TO significantly. Performance of a pringle maneuver was associated with higher odds of achieving TO (OR = 8.35 (1.35–51.48), *p* = 0.022), which is remarkable as it is not an independent predictor of reduced blood loss. Major complications were significantly more likely after multivisceral resections with an OR = 20.1 (2.48–163.0).

As some of the predictors, e.g., OR for multivisceral resection and pringle maneuver, were associated with wide confidence intervals, this may indicate substantial uncertainty.

IWATE score, major liver resection, liver re-resection, and status of previous abdominal surgery were not independently associated with any of the outcome parameters, while the robotic approach only significantly reduced blood loss.

### 3.5. Quality of Life

Patient-reported outcomes assessed using the EQ-5D-5L system were comparable between surgical approaches, including the assessment of overall health status via visual analogue scale (0–100). The median score was 74.5 (50.0–85.0) for patients undergoing open surgery and 79.5 (59.0–84.0) for those undergoing robotic surgery (*p* = 0.508). The MD in visual analogue scores between the two groups was −4.23 (−14.02–5.56), indicating that there was neither a clinically nor a statistically significant difference in self-rated overall health status between the surgical approaches. Across all five dimensions (mobility, self-care, usual activities, pain/discomfort, and anxiety/depression), no significant overall differences were observed between patients undergoing open or robotic liver surgery (all *p*-values > 0.10).

### 3.6. Propensity Score Matching

Propensity score matching resulted in 33 matched pairs (*n* = 66). Prior to matching, substantial baseline differences were observed, particularly in Charlson Comorbidity Index (standardized mean difference (SMD) 0.39), liver re-resection (SMD 0.21), and age (SMD −0.26), indicating a tendency toward more complex and comorbid patients undergoing open surgery. After matching, balance was markedly improved across 12 of 14 covariates (SMD < 0.1; see Supplementary Material S1). Residual imbalance persisted for age (SMD −0.11) and Charlson Comorbidity Index (SMD −0.22), although both were substantially reduced compared with the unmatched cohort. Detailed results are presented in Table 4.

With regard to complication severity according to CDC, grade IIIb complications occurred significantly more frequently in the robotic group (21.2% vs. 0.0%; *p* = 0.011), whereas no significant differences were found for major complications in total. Rates of bile leak, PHLF, and PHH were comparable between groups and in ISGLS subgroups, the latter being limited by very small event number. Likewise, no significant differences were observed in resection margin status. In a sensitivity analysis, bile duct reconstruction was additionally included in the propensity score model. This resulted in 30 matched pairs. Covariate balance remained acceptable for most variables, and bile duct reconstruction itself was fully balanced after matching (SMD 0.0; see Supplementary Material S1), whereas minor residual imbalance persisted for Charlson Comorbidity Index (SMD 0.11) and multivisceral resection (SMD 0.1). Overall, inclusion of biliodigestive anastomosis did not materially change the balance pattern, thus supporting the robustness of the main matching strategy.

Outcome analyses were additionally adjusted for both variables (Table 5): Robotic surgery remained associated with lower blood loss (adjusted mean difference: −309 mL, 95% CI from −533 to −86; *p* = 0.009), whereas no statistically significant associations were observed for operative duration (−11 min; 95% CI from −45 to 24; *p* = 0.535), LOHS (0.08 days; 95% CI from −6.76 to 6.92; *p* = 0.982), Textbook Outcome (OR 0.94; 95% CI 0.28–3.18; *p* = 0.925), or major complications (OR 1.43; 95% CI 0.37–5.52; *p* = 0.607).

### 3.7. Learning Curve Analysis

As mentioned in the Methods Section, no individual learning curve analysis was performed. Generally speaking, one of the surgeons was trained at an academic center with previous experience in robotic liver surgery. At the institution, there was no former experience with robotic liver surgery, and the experienced surgeon was involved in all resections.

**Figure 1:**
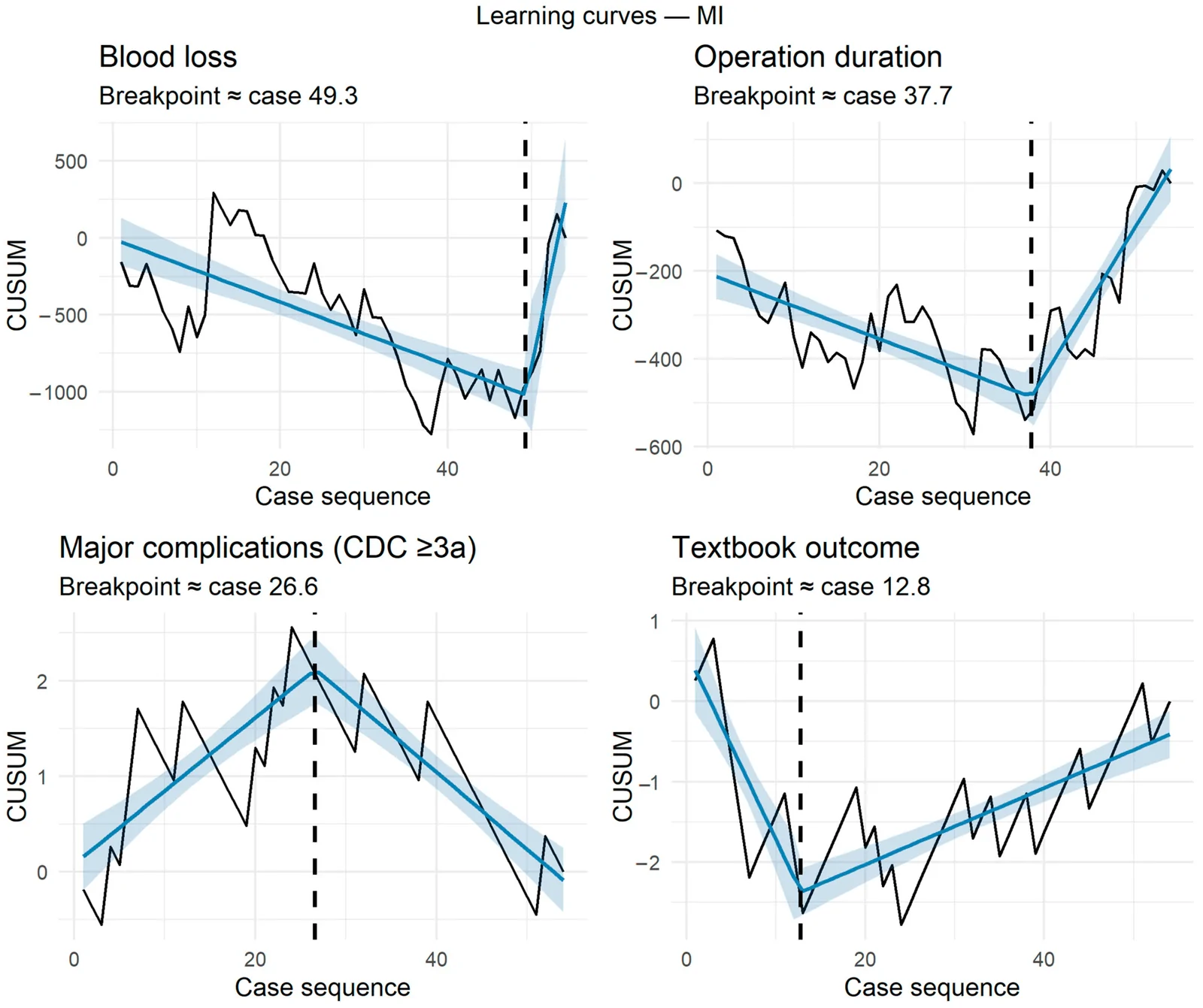
Unadjusted cumulative sum (CUSUM) learning curves for estimated blood loss, operative duration, major complications and TO according to chronological robotic case sequences. Dashed vertical lines indicate estimated breakpoints.

In the robotic cohort, learning curves demonstrated outcome-specific breakpoints: blood loss (breakpoint: 49.3 cases (47.3–49.3; *p* < 0.001)) and operation duration (breakpoint: 37.7 cases (35.4–37.7; *p* < 0.001)) decreased until reaching their breakpoint and increased again afterwards (see Figure 1). In contrast, the rate of major complications peaked at case 26.6 (23.4–26.6; *p* <0.001) corresponding to approximately two more major complications than expected, and declined afterwards (see Figure 1). In concordance, the composite parameter TO improved after reducing its probability until 12.8 cases (10.4–12.8 cases; *p* < 0.001; modest underperformance of approximately 2.5 fewer TO cases than expected at breakpoint; see Figure 1).

All identified breakpoints were statistically significant, supporting the multidimensional nature of surgical learning.

### 3.8. Adjustment for IWATE Levels

**Figure 2:**
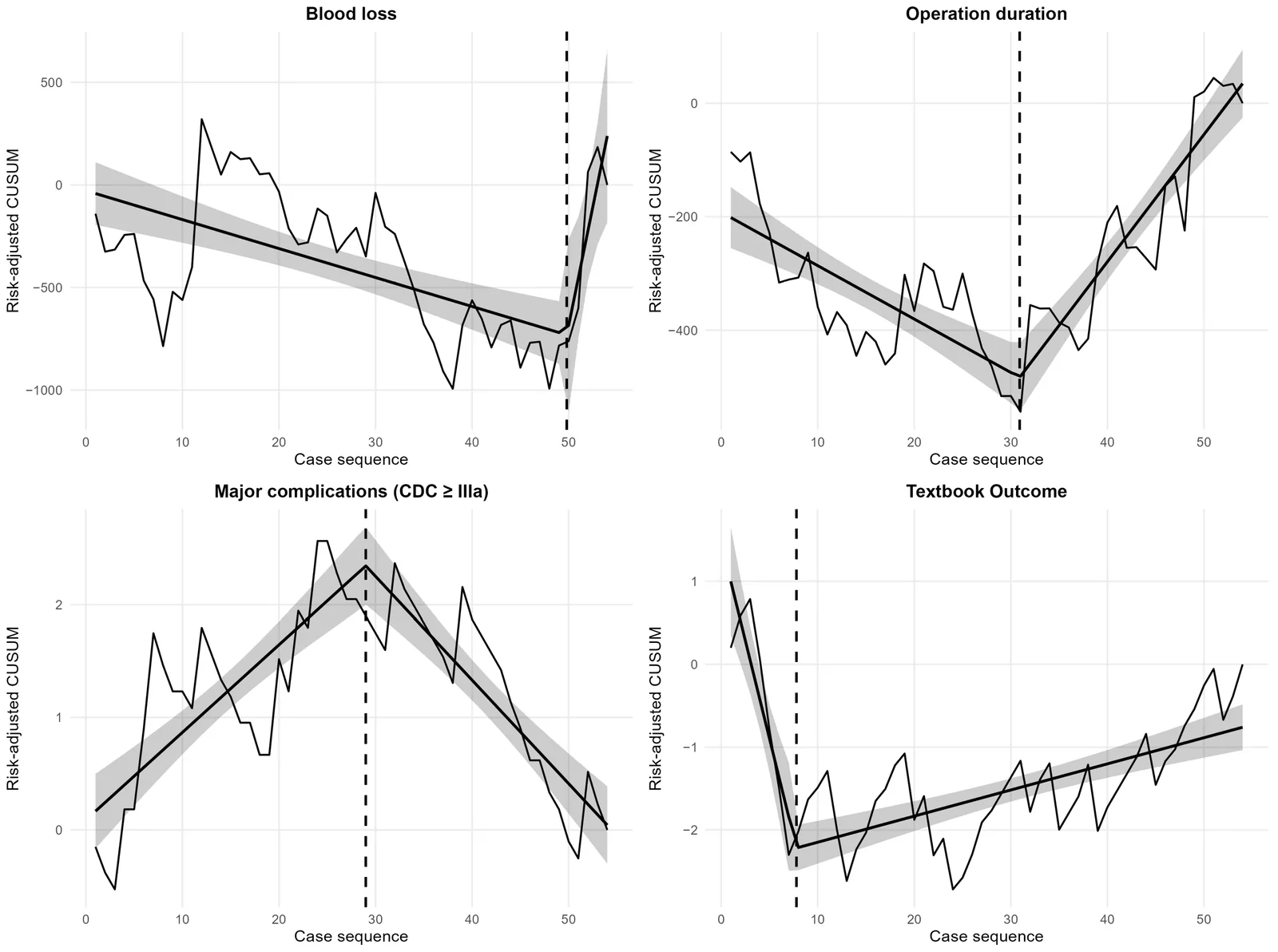
IWATE difficulty level-adjusted cumulative sum (CUSUM) learning curves for estimated blood loss, operative duration, major complications and TO according to chronological robotic case sequence. Dashed vertical lines indicate estimated breakpoints; shaded areas represent the fitted segmented regression curves with uncertainty intervals.

After adjustment for IWATE difficulty levels, all breakpoints remained statistically significant (Figure 2): The breakpoint for blood loss occurred after 49.8 cases (14.1–50.6); *p* = 0.004), which is similar to the one before adjustment (49.3 cases). For blood loss, the change-point was estimated late in the sequence with a wide CI, suggesting a gradual rather than abrupt improvement or an edge-effect of single change-point modelling. Operation duration changed at 30.9 cases (27.7–35.1); *p* < 0.001), earlier than before adjustment (37.7 cases). Additionally, the breakpoint for major complications occurred after 29 cases (25.3–37.5; *p* < 0.001), which is close to 26.6 cases before adjustment. TO breakpoint, comparable to the calculation before IWATE-level-adjustment, occurred the earliest (7.8 cases (7.0–25.0); *p* < 0.001).

Confidence intervals were wide for some outcomes—particularly blood loss and major complications—indicating uncertainty in the exact breakpoint location despite evidence for a slope change. Nevertheless, the learning curves were broadly similar before and after adjustment (Figure 1 and Figure 2).

### 3.9. Economic Subgroup Analysis

A total of 48 patients underwent open and 34 undergoing robotic liver surgery. The two subgroups were comparable regarding age, BMI, CCI, major complications, and TO, while estimated intraoperative blood loss, duration of surgery, as well as LOHS were significantly lower in the robotic group. Charlson Comorbidity Index was significantly increased in the open group (6.00 (4.00–6.00) vs. 5.00 (2.00–6.00); *p* = 0.035), while a trend towards lower IWATE scores was observed in the robotic group (8 (6–10) vs. 6 (5–9); *p* = 0.095).

Total costs did not differ significantly between both groups: EUR 14,805.48 (11,935.58–20,002.90) vs. EUR 15,380.44 (11,357.20–21,117.37); *p* = 0.944. Intraoperative costs were significantly higher in the robotic group (EUR 6637.11 (4851.77–8360.87) vs. EUR 10,155.97 (8769.55–13,117.46); *p* < 0.001), while inpatient costs were significantly lower after robotic resection (EUR 6573.99 (5279.94–11,059.61) vs. EUR 4904.07 (3183.02–9386.79); *p* = 0.035). Treatment-related costs, including diagnostics and interventions, did not differ significantly.

In stratified multivariable linear models for total costs, major complications were independently associated with higher costs in both approaches (EUR 33,870.59 (16,764.52–50,976.66; *p* < 0.001) and EUR 22,621.58 (2428.99–42,814.17; *p* = 0.029)), but in open surgery, higher IWATE score (EUR 1993.41 per point (164.17–3822.65); *p* = 0.033) and increasing blood loss (EUR 4849.06 per 100 mL (2156.85–7541.28); *p* = 0.001) in the robotic group were significant, independent cost drivers.

Higher Charlson Comorbidity Index was associated with increased net value (β = EUR 1151 per point (213–2089); *p* = 0.017), likely reflecting higher reimbursement in more complex patients, as well as TO (β = EUR 5823 (741–10,905); *p* = 0.025). In contrast, prolonged LOHS was independently associated with a significant reduction in net value (β = EUR -393 per day (−615–−172); *p* < 0.001), representing the strongest negative economic driver in the model.

Operative complexity (IWATE score, operative duration, blood loss), surgical approach, and performance of major resection were not independently associated with net value after adjustment for other covariates. These findings suggest that postoperative efficacy and achievement of optimal clinical outcomes exert a greater influence on economic performance than intraoperative technical factors or surgical approach alone.

## 4. Discussion

This study evaluates the implementation process of a robotic liver surgery, with generally similar outcomes to open surgery across clinical, technical, economic, and patient-reported quality-of-life domains. Shared decision making on the choice of approach had not been documented, but was reasoned on clinical characteristics, patients’ preferences, and availability of the robotic system in general. As significance of shorter operation duration and tendencies towards fewer liver re-resections, multivisceral resections, and prior abdominal surgery were observed in robotic surgery, a selection bias beyond technical complexity, illustrated by comparable IWATE scores, is possible. This could explain significantly shorter operation duration and less blood loss; therefore, propensity score matching was performed: While imbalance remained for age and comorbidity index, key indicators of surgical complexity, including IWATE level and extent of resection, were well balanced, suggesting adequate control of procedure-related confounding. Because of sparse events in the ISGLS subgroups, these level-wise analyses were exploratory and should be interpreted cautiously. After adjusting PSM for age and Charlson Comorbidity Index, robotic surgery was persistently associated with significantly reduced blood loss, while differences in TO, major complications, operation duration, and LOHS were not significant between both groups. These findings align with previous reports indicating that early adoption of robotics does not compromise perioperative safety or quality of recovery [5,7,8,9,10,11,12,17,29]. Meanwhile, in (established) robotic living donor hepatectomy programs, not only blood loss but also major complications were significantly reduced compared to the open approach group [30]. The findings of multivariable analysis should be interpreted with care due to wide confidence intervals implying larger uncertainty. A notable finding was the higher rate of CDC IIIb, in concordance with non-significant increase in concordant reoperations and grade C bile leakages in the robotic group. Although the overall rate of major complications did not differ significantly between approaches, the small number of events and wide confidence interval of the adjusted estimate preclude conclusions regarding equivalence or comparative safety. These findings may reflect procedural selection and learning effects during implementation but should be interpreted cautiously given the limited sample size.

Learning curve analysis revealed outcome-specific trajectories, highlighting the multidimensional nature of surgical proficiency. Technical parameters like blood loss and duration of surgery improved until reaching their (late) breakpoints at 49.3 cases and 37.7 cases, respectively, while major complication rate peaked at 26.6 cases and declined afterwards. These results are broadly consistent with current guidelines recommending the performance of at least 25 major and 15 minor robotic liver resections [14]. Yet, due to small sample size, wide confidence intervals, and edge effect of late breakpoint manifestation, breakpoints should be interpreted as exploratory status. Dugan et al. observed breakpoints at 75 minor and 100 major resections, requiring an additional 57 complex minor resections for high-complexity mastery [10]. Additionally, as major complication rate declined after 26.6 cases, it finally reached its initial set point, which is within the four-phase progression across 100 resections proposed by Rahimli et al. ranging from technical competency (1–20) to “full mastery” (66–100) [19].

Adjustment for procedural complexity using IWATE levels resulted in similar curve patterns, indicating that increasing case difficulty did not substantially bias learning curve interpretation. However, wide confidence intervals—particularly for blood loss and major complications—suggest uncertainty in the exact timing of breakpoints and support the concept of a gradual rather than abrupt transition in proficiency (see Figure 1 and Figure 2). This indicates that “full mastery” [19] has not been reached within 54 cases as technical outcomes increased after 30.9 and 49.3 cases, respectively. Notably, some studies report significant reductions in major complications [5,10,31] or conversion rate [5,7] during implementation process. Our conversion rate (2/54 robotic cases) is comparable to that reported elsewhere [5,11,17,30]. TO has not been established as composite clinical outcome parameter during robotic implementation process to date; in our complexity-adjusted analysis, it reached its lower turning point the earliest (after 7.8 cases; Figure 2), underlining its importance by suggesting that composite endpoints may detect performance shifts earlier than isolated technical metrics [23].

From an economic perspective, both approaches demonstrated similar total hospital costs. The substantially higher intraoperative costs in the robotic group were balanced by lower ICU and general ward costs. These findings are in line with previous analyses [16,30,31,32]. A higher Charlson Comorbidity Index was a predictor of positive net value, whereas a prolonged LOHS was a predictor of negative net value. In concordance to our findings, major complications, particularly PHLF grades B/C, are consistently identified as the strongest economic cost driver [33,34,35]. Our economic subgroup analysis reinforces this: complications drive costs.

Quality of life at 90 days after resection was comparable between both groups (74.5 (50.0–85.0) vs. 79.5 (59.0–84.0)), which is within the range of minimum clinically important differences after surgery [36]. Additionally, these minimum clinically important differences differ between populations and gender [37]. This suggests that the robotic approach does not adversely affect patient-reported recovery during the early implementation phase.

As the aim of this study was to focus on safety and efficacy of the implementation process of a robotic liver program, we focused on comparing the robotic approach to the established open approach. Therefore, laparoscopic patterns were not taken into account.

### Limitations

The study is limited by its small sample size, its retrospective design and its non-normal distribution, particularly within the economic subset (2023–2024). Due to the retrospective nature of this study, there was no protocolized decision regarding the choice of approach and its reasons. Selection bias is likely, as more complex operations and especially re-resections were more frequently managed via open approach. Learning curve models should be interpreted with care regarding potential edge effects as some breakpoints manifest at the end of case series. Due to anonymization, surgeon-level effects could not be evaluated, which contrasts with studies incorporating individual learning trajectories [38,39]. Economic results from a single large municipal hospital may not be generalized to other health systems.

## 5. Conclusions

Implementation of a robotic liver surgery program was not associated with significant differences in major complications, TO, total costs, or quality of life compared with open surgery, while blood loss was lower after adjustment, although the reasons for the individual choice of approach had not been documented. The late changes in major complication rate, blood loss, and duration of surgery provided reason for an unfinished learning curve within the study period. Major complications—not surgical approach—were the independent risk factor for increasing total costs. TO was found to be the most informative composite endpoint, reaching its lower turning point at case 7.8 after adjustment for IWATE level.

Further prospective multicenter studies are required to validate these findings and to define learning phases in robotic liver surgery and validate economic implications across different healthcare systems.

## Figures and Tables

**Table 1:** Preoperative clinical and pathological characteristics.

	Open *n* = 54 (50%)	Robotic *n* = 54 (50%)	MD or OR with 95%-CI in ()	*p*-Value
Age (years)	63.5 (51–72)	68 (61–74)	MD = −3.31 (−8.27–1.64)	0.153
Gender f/m (*n*)	19 (35.2%)/35 (64.8%)	21 (38.9%)/33 (61.1%)	OR = 1.17 (0.54–2.56)	0.842
BMI (kg/m^2^)	24.72 (IQR: 22.22–29.35)	25.86 (IQR: 23.81–30.49)	MD = −1.27 (−3.50–0.97)	0.167
ASA Score				
II	11 (20.4%)	12 (22.2%)	OR = 0.90 (0.36–2.25)	>0.99
III	42 (77.8%)	42 (77.8%)	OR = 1.00 (0.40–2.48)	>0.99
IV	1 (1.9%)	0 (0%)		>0.99
Charlson Comorbidity Index	6 (IQR: 4–6)	5.5 (IQR: 2–6)	MD = 0.83 (0.01–1.66)	**0.049**
Pathological Characteristics				
Colorectal liver metastases	25 (46.3%)	13 (24.1%)	OR = 2.72 (1.20–6.18)	**0.027**
Hepatocellular carcinoma (HCC)	11 (20.4%)	19 (35.2%)	OR = 0.47 (0.20–1.12)	0.133
Intrahepatic cholangiocarcinoma (iCC)	5 (9.3%)	7 (13.0%)	OR = 0.69 (0.20–2.31)	0.759
Gallbladder carcinoma	3 (5.6%)	1 (1.9%)	OR = 3.12 (0.31–30.96)	0.61
Neuroendocrine tumor metastasis	0 (0.0%)	1 (1.9%)	OR = 0.33 (0.01–8.21)	>0.99
Other liver metastases	4 (7.4%)	5 (9.3%)	OR = 0.78 (0.20–3.09)	>0.99
Hepatic adenoma	0 (0.0%)	3 (5.6%)	OR = 0.13 (0.01–2.68)	0.242
Hemangioma	1 (1.9%)	1 (1.9%)	OR = 1.00 (0.06–16.41)	>0.99
Focal nodular hyperplasia (FNH)	0 (0.0%)	1 (1.9%)	OR = 0.33 (0.01–8.21)	>0.99
Other benign lesions	5 (9.3%)	3 (5.6%)	OR = 1.73 (0.39–7.65)	0.713

ASA = American Society of Anesthesiologists; BMI = body mass index; MD = mean difference; OR = odd’s ratio; 95%-CI = 95% confidence interval; IQR = interquartile range. All significant results are marked in bold letters.

**Table 2:** Procedural characteristics.

	Open Approach (*n* = 54)	Robotic Approach (*n* = 54)	MD or OR with 95%-CI in ()	*p*-Value
Major Resection	17 (31.5%)	12 (22.2%)	OR = 1.61 (0.68–3.80)	0.385
Atypical Resection	28 (51.9%)	24 (44.4%)	OR = 1.35 (0.63–2.87)	0.563
Anatomical Segmentectomy	14 (25.9%)	17 (31.5%)	OR = 0.76 (0.33–1.76)	0.671
Bisegmenectomy (II + III)	2 (3.7%)	3 (5.6%)	OR = 0.65 (0.10–4.08)	>0.99
Posterior Sectionectomy (VI + VII)	2 (3.7%)	1 (1.9%)	OR = 2.04 (0.18–23.17)	>0.99
Left Hemihepatectomy (II–IV)	4 (7.4%)	4 (7.4%)	OR = 1.00 (0.24–4.22)	>0.99
Right Hemihepatectomy (V–VIII)	9 (16.7%)	6 (11.1%)	OR = 1.60 (0.53–4.86)	0.578
Left Trisectionectomy (I–V + VIII)	0 (0%)	0 (0%)	Not available	Not available
Right Trisectionectomy (IV–VIII)	3 (5.6%)	0 (0%)	OR = 7.41 (0.37–146.96)	0.242
Mesohepatectomy (IV + V + VIII)	0 (0%)	1 (1.9%)	OR = 0.33 (0.01–8.21)	>0.99
ALPPS	1 (1.9%)	3 (5.6%)	OR = 0.32 (0.03–3.19)	0.61
Other types of resection	7 (13%)	1 (1.9%)	OR = 7.89 (0.94–66.54)	0.066
Multivisceral resection	11 (20.4%)	5 (9.3%)	OR = 2.51 (0.81–7.79)	0.176
Liver Re-resection	15 (27.8%)	4 (7.4%)	OR = 4.81 (1.48–15.64)	**0.011**
Major previous surgery	25 (46.3%)	15 (28.3%)	OR = 2.24 (1.01–4.99)	0.085
Bile duct reconstruction/biliodigestive anastomosis	3 (5.6%)	0 (0%)	OR = 7.41 (0.37–146.96)	0.242
IWATE Score	7.00 (IQR: 6–10)	6.50 (IQR: 5–10)	MD = 0.75 (−0.28–1.79)	0.154
Pringle’s maneuver	10 (18.5%)	14 (25.9%)	OR = 0.65 (0.26–1.63)	0.487
IWATE level				
Low	1 (1.9%)	7 (13.0%)	OR = 0.13 (0.02–1.09)	0.070
Intermediate	18 (34.0%)	20 (37.0%)	OR = 0.87 (0.40–1.93)	0.896
Advanced	16 (30.2%)	13 (24.1%)	OR = 1.36 (0.58–3.21)	0.621
Expert	18 (34.0%)	14 (25.9%)	OR = 1.47 (0.64–3.38)	0.486
Overall				0.144
Duration (min)	209 (IQR: 160–264)	166.5 (IQR: 130–216)	MD = 31.57 (2.17–60.98)	**0.014**
Estimated intraoperative blood loss (mL)	400 (IQR: 150–850)	100 (IQR: 50–300)	MD = 385.28 (220.68–549.88)	**<0.001**
Dichotomized intraoperative transfusion of:				
red blood cell concentrates	13 (24.1%)	2 (3.7%)	OR = 8.24 (1.76–38.60)	**0.0054**
thrombocyte concentrates	1 (1.9%)	0 (0.0%)	OR = 3.06 (0.122–76.48)	>0.99
Fresh Frozen Plasma	15 (27.8%)	5 (9.3%)	OR = 3.77 (1.26–11.27)	**0.0258**

ALPPS = associating liver partition with portal vein ligation staged hepatectomy; MD = mean difference; OR = odd’s ratio; 95%-CI = 95% confidence interval; IQR = interquartile range. Intraoperative transfusions are presented as dichotomized variables indicating the presence or absence of transfusion and were derived from the original continuous transfusion variables. All significant results are marked in bold letters.

**Table 3:** Clinical outcomes of the open and robotic approaches.

	Open Approach (*n* = 54)	Robotic Approach (*n* = 54)	MD or OR with 95% Confidence Interval in ()	*p*-Value
Textbook Outcome	37 (68.5%)	40 (74.1%)	OR = 0.76 (0.33–1.76)	0.671
Complications according to CDC:				Overall: 0.349
No complications	35 (64.8%)	36 (66.7%)	OR = 0.92 (0.42–2.04)	>0.99
Grade I	11 (21.2%)	8 (15.1%)	OR = 1.51 (0.55–4.12)	0.58
Grade II	8 (15.7%)	9 (17.0%)	OR = 0.91 (0.32–2.58)	>0.99
Grade IIIa	6 (11.5%)	2 (3.8%)	OR = 3.33 (0.64–17.31)	0.258
Grade IIIb	0 (0%)	7 (13.2%)	OR = 0.057 (0.003–1.025)	0.018
Grade IVa	3 (5.8%)	1 (1.9%)	OR = 3.12 (0.31–31.05)	0.61
Grade IVb	0 (0%)	3 (5.6%)	OR = 0.13 (0.01–2.56)	0.235
Grade V	3 (5.6%)	2 (3.7%)	OR = 1.53 (0.25–9.53)	>0.99
Major complications (CDC ≥ IIIa)	9 (16.7%)	10 (18.5%)	OR = 0.88 (0.33–2.37)	>0.99
Comprehensive Complication Index	0.00 (IQR: 0.00–20.90)	0.00 (IQR: 0.00–20.90)	MD = 0.51 (−9.47–10.50)	0.761
Reoperation	4 (7.4%)	8 (14.8%)	OR = 0.46 (0.13–1.63)	0.358
PHLF	4 (7.4%)	3 (5.6%)		Overall: 0.907
Grade A	1 (1.9%)	1 (1.9%)	OR = 0.67 (0.02–18.06)	>0.99
Grade B	2 (3.7%)	1 (1.9%)	OR = 2.00 (0.09–44.35)	>0.99
Grade C	1 (1.9%)	1 (1.9%)	OR = 0.67 (0.02–18.06)	>0.99
PHH	4 (7.4%)	0 (0%)		Overall: 0.126
Grade A	0 (0%)	0 (0%)	OR = 0.11 (0.00–14.76)	Not applicable
Grade B	1 (1.9%)	0 (0%)	OR = 0.43 (0.01–33.60)	Not applicable
Grade C	3 (5.6%)	0 (0%)	OR = 2.33 (0.03–182.92)	Not applicable
Bile leakage	4 (7.4%)	2 (3.7%)		Overall: 0.674
Grade A	0 (0%)	0 (0%)	OR = 0.56 (0.01–37.57)	Not applicable
Grade B	4 (7.4%)	0 (0%)	OR = 45.00 (0.67–3042.81)	0.126
Grade C	0 (0%)	2 (3.7%)	OR = 0.02 (0.00–1.50)	0.126
LOHS (days)	7.00 (IQR: 6–12)	5.00 (IQR: 4–9)	MD = 2.44 (−2.08–6.97)	<0.0001
30 day readmission	5 (9.3%)	3 (5.6%)	OR = 1.73 (0.39–7.65)	0.713
R-status				Overall: 0.947
R0	44 (84.6%)	46 (86.8%)	OR = 0.84 (0.28–2.50)	0.968
R1	5 (9.6%)	5 (9.4%)	OR = 1.02 (0.28–3.76)	>0.99
R2	1 (1.9%)	1 (1.9%)	OR = 1.02 (0.06–16.74)	>0.99
Rx	2 (3.7%)	1 (1.9%)	OR = 2.08 (0.18–23.67)	0.987
Missing	2 (3.7%)	1 (1.9%)		

CDC = Clavien–Dindo classification; LOHS = length of hospital stay; MD = mean difference; OR = odd’s ratio; IQR = interquartile range; PHH = posthepatectomy hemorrhage; PHLF = posthepatectomy liver failure. All significant results are marked in bold letters.

**Table 4:** Baseline characteristics, operative variables, and postoperative outcomes after propensity score matching.

	Open Approach	Robotic Approach	*p*-Value
Baseline characteristics			
Age (years)	64 (IQR: 60–71)	67 (IQR: 60–74)	0.416
Gender f/m (*n*)	14 (42.4%)/19 (57.6%)	15 (45.5%)/18 (54.5%)	>0.99
BMI (kg/m^2^)	25.73 (23.24–30.93)	25.35 (23.46–29.54)	0.844
Charlson Comorbidity Index	6 (4–6)	6 (5–6)	0.302
IWATE score	7 (6–10)	8 (5–10)	0.927
ASA Score			
ASA II (*n*)	7 (21.2%)	5 (15.2%)	0.75
ASA III (*n*)	26 (78.8%)	28 (84.8%)	0.75
Cirrhosis (*n*)	8 (24.2%)	7 (21.2%)	>0.99
Intraoperative characteristics			
Multivisceral resection (*n*)	5 (15.2%)	5 (15.2%)	>0.99
Bile duct anastomosis	2 (6.1%)	0 (0.0%)	0.473
Liver re-resection	5 (15.2%)	4 (12.1%)	>0.99
Major resection	12 (36.4%)	10 (30.3%)	0.794
Blood loss (mL)	350 (IQR: 150–800)	200 (IQR: 50–400)	**0.032**
Operative duration (min)	214 (IQR: 156–263)	171 (IQR: 133–230)	0.243
Postoperative outcomes			
LOHS (days)	7 (IQR: 6–11)	6 (IQR: 5–15)	0.68
Comprehensive Complication Index	0 (0–20.9)	0 (0–34.6)	0.6
EQ-5D VAS	60 (41–82)	77 (50–81)	0.554
Textbook Outcome	24 (72.7%)	22 (66.7%)	0.789
Complications (CDC) (*n*)			
Grade I	5 (15.2%)	5 (15.2%)	>0.99
Grade II	5 (15.2%)	7 (21.2%)	0.751
Grade IIIa	3 (9.1%)	1 (3.0%)	0.613
Grade IIIb	0 (0.0%)	7 (21.2%)	**0.011**
Grade IVa	2 (6.1%)	1 (3.0%)	>0.99
Grade IVb	0 (0.0%)	3 (9.1%)	0.238
Grade V	2 (6.1%)	2 (6.1%)	>0.99
Major complications (CDC ≥ IIIa; *n*)	6 (18.2%)	9 (27.3%)	0.557
Bile leakage	3 (9.1%)	2 (6.1%)	>0.99
Grade A	0 (0.0%)	0 (0.0%)	>0.99
Grade B	3 (9.1%)	0 (0.0%)	0.1
Grade C	0 (0.0%)	2 (6.1%)	0.1
PHLF	3 (9.1%)	3 (9.1%)	>0.99
Grade A	1 (3.0%)	1 (3.0%)	>0.99
Grade B	2 (6.1%)	1 (3.0%)	>0.99
Grade C	0 (0.0%)	1 (3.0%)	>0.99
PHH	3 (9.1%)	0 (0.0%)	0.248
Grade A	0 (0.0%)	0 (0.0%	>0.99
Grade B	0 (0.0%)	0 (0.0)	>0.99
Grade C	3 (9.1%)	0 (0.0%)	>0.99
R-status			
R0	29 (90.6%)	27 (84.4%)	0.708
R1	1 (3.1%)	4 (12.5%)	0.355
R2	1 (3.1%)	1 (3.1%)	>0.99
RX	1 (3.1%)	0 (0.0%)	>0.99

CDC = Clavien–Dindo classification; LOHS = length of hospital stay; IQR = interquartile range; PHH = posthepatectomy hemorrhage; PHLF = posthepatectomy liver failure. Continuous variables are presented as median (IQR) and categorical variables as *n* (%). Significant results are marked in bold letters.

**Table 5:** Clinical outcomes after propensity score matching and additional adjustment for residual covariate imbalance.

Outcome	Open Approach (*n* = 33)	Robotic Approach (*n* = 33)	Adjusted Effect (95% CI)	*p*-Value
Blood loss (mL)	350 (IQR 150–800)	200 (IQR 50–400)	MD −309 mL (−533 to −86)	**0.009**
Operative duration (min)	214 (IQR 156–263)	171 (IQR 133–230)	MD −11.0 min (−45.4 to 23.5)	0.535
LOHS (days)	7 (IQR 6–11)	6 (IQR 5–15)	MD +0.08 days (−6.76 to 6.92)	0.982
Textbook Outcome	24 (72.7%)	22 (66.7%)	OR 0.94 (0.28–3.18)	0.925
Major complications (CDC ≥ IIIa)	6 (18.2%)	9 (27.3%)	OR 1.43 (0.37–5.52)	0.607

Outcome analyses after propensity score matching were adjusted for age and Charlson Comorbidity Index because these variables showed residual imbalance. Regression models used cluster-robust standard errors accounting for matched pairs. CDC = Clavien–Dindo classification; LOHS = length of hospital stay; MD = Mean differences. Continuous variables are presented as median (IQR) and categorical variables as *n* (%). Significant results are marked in bold letters.

## Data Availability

The data presented in this study are available upon request from the corresponding author.

## Supplementary Materials

The following supporting information can be downloaded at: https://www.mdpi.com/article/10.3390/jcm15166413/s1, Supplementary Material S1: Standardized mean differences (SMDs) before and after PSM.

## Abbreviations

The following abbreviations are used in this manuscript:

95%-CI	95%-confidence interval
ASA	American Society of Anesthesiologists—Physical Status Classification
BMI	Body Mass Index [kg/m^2^]
CDC	Clavien–Dindo classification
CCI	Comprehensive Complication Index
CUSUM	Cumulative Sum
HCC	Hepatocellular Carcinoma
iCC	Intrahepatic Cholangiocarcinoma
IQR	Interquartile Range
LOHS	Length of hospital stay
MD	Mean Difference
OR	Odd’s Ratio
BL	Posthepatectomy bile leakage
PHH	Posthepatectomy hemorrhage
PHLF	Posthepatectomy liver failure
R-Status	Tumor residual status
SD	Standard Deviation
TO	Textbook Outcome
EQ-5D-5L	5-level EQ-5D version
