# Supplementary material for: Implementation of a Robotic Liver Surgery Program in a Large Municipal Hospital: A Propensity Score-Matched Analysis of Clinical, Economic, and Learning Curve Outcomes
Source: J Clin Med. 2026 Aug 19;15(16):6413. doi: 10.3390/jcm15166413 (PMC13513414; doi:10.3390/jcm15166413)
Supplement: Supplementary file 1 [file jcm-15-06413-s001.zip › jcm-4454823-supplementary.pdf]

Supplementary Material S1

Supplementary Material S1: Standardized mean differences (SMD) before and after PSM

Covariate	SMD before matching	SMD after matching	SMD after BDA sensitivity analysis
Age	-0.26	-0.11	-0.035
BMI	-0.21	0.08	-0.090
ASA 2	-0.01	0.06	0.033
ASA 3	-0.00	-0.06	-0.033
ASA 4	0.02	0.00	0.000
Charlson Comorbidity Index	0.39	-0.22	0.111
IWATE level 1	-0.11	-0.06	0.033
IWATE level 2	-0.03	0.06	-0.033
IWATE level 3	0.06	0.03	0.033
IWATE level 4	0.08	-0.03	-0.033
Multivisceral	0.11	0.00	0.100
Liver re-resection	0.21	0.03	0.000
Cirrhosis	-0.09	0.03	-0.100
Major resection	0.10	0.06	-0.033
Bile duct anastomosis	0.06		0.000

SMD < 0.10 were considered indicative of adequate balance. After matching, 12 of 14 covariates were balanced. Residual imbalance remained for age and Charlson Comorbidity Index. After sensitivity analysis including biliodigestive anastomosis (BDA), 30 matched pairs were obtained. Residual imbalance remained for Charlson Comorbidity Index and multivisceral resection, whereas BDA and liver re-resection were fully balanced after matching.
